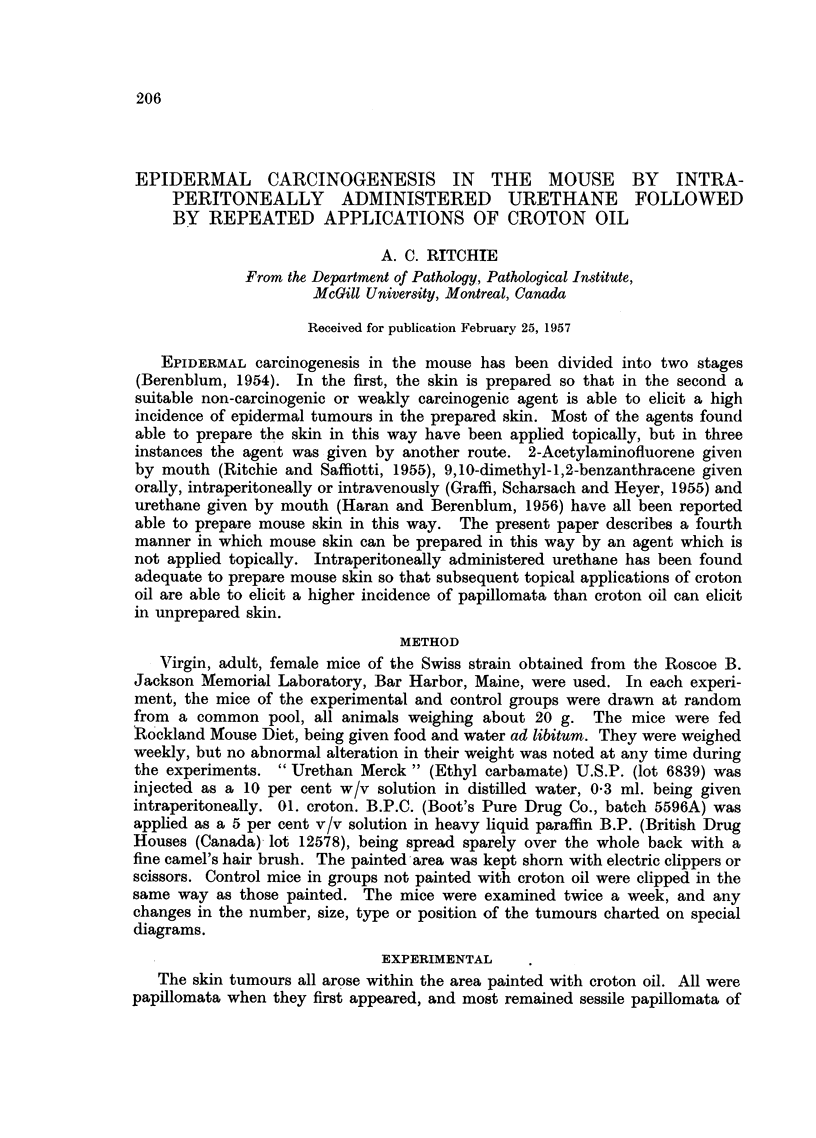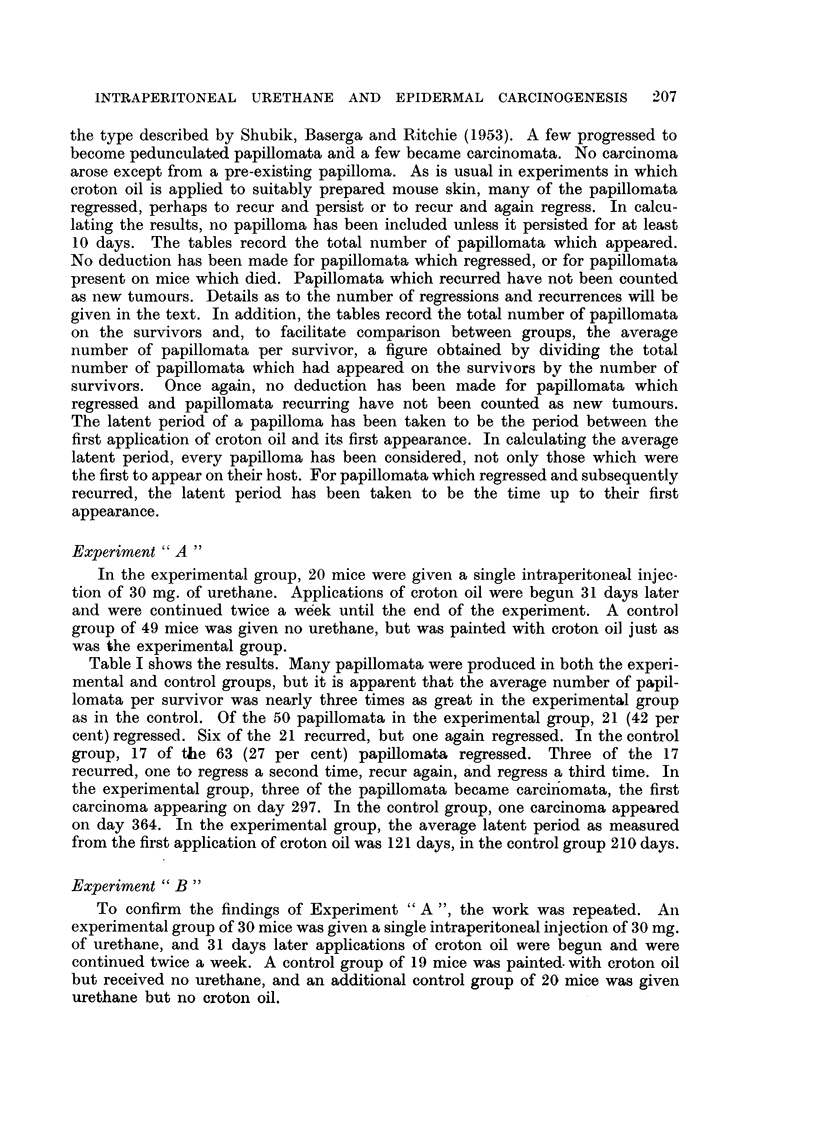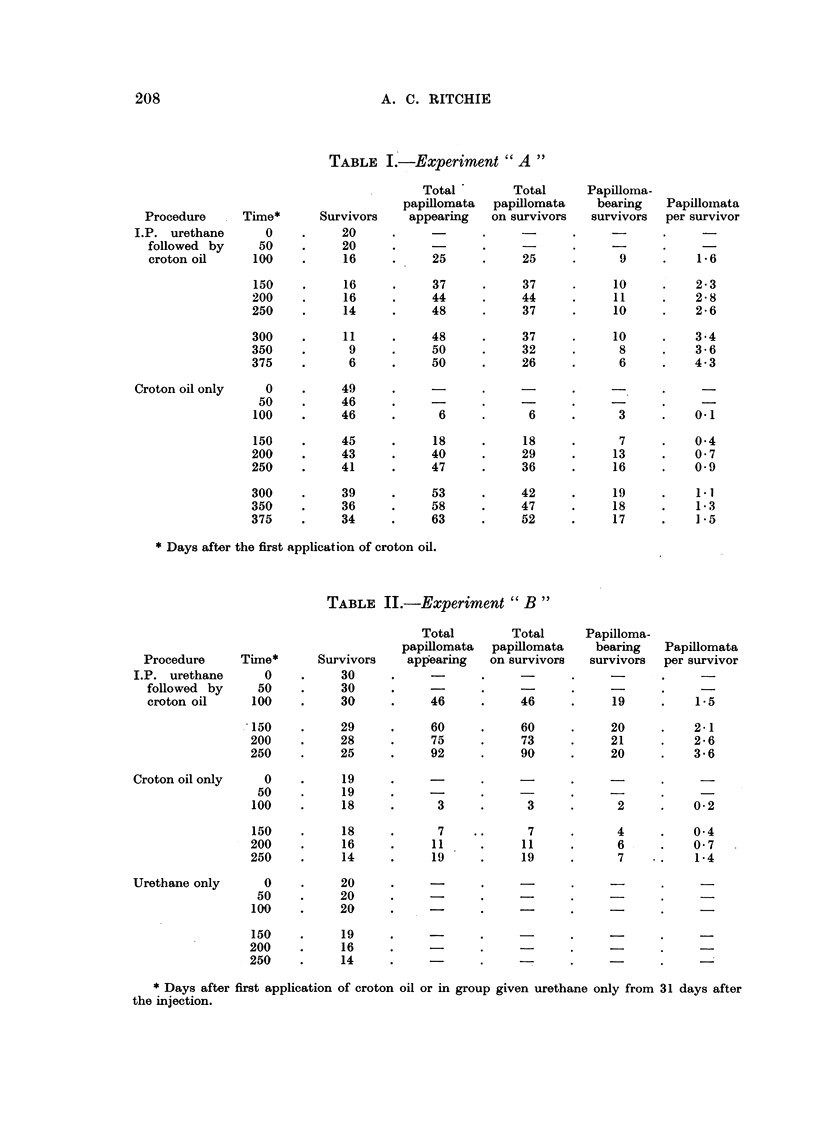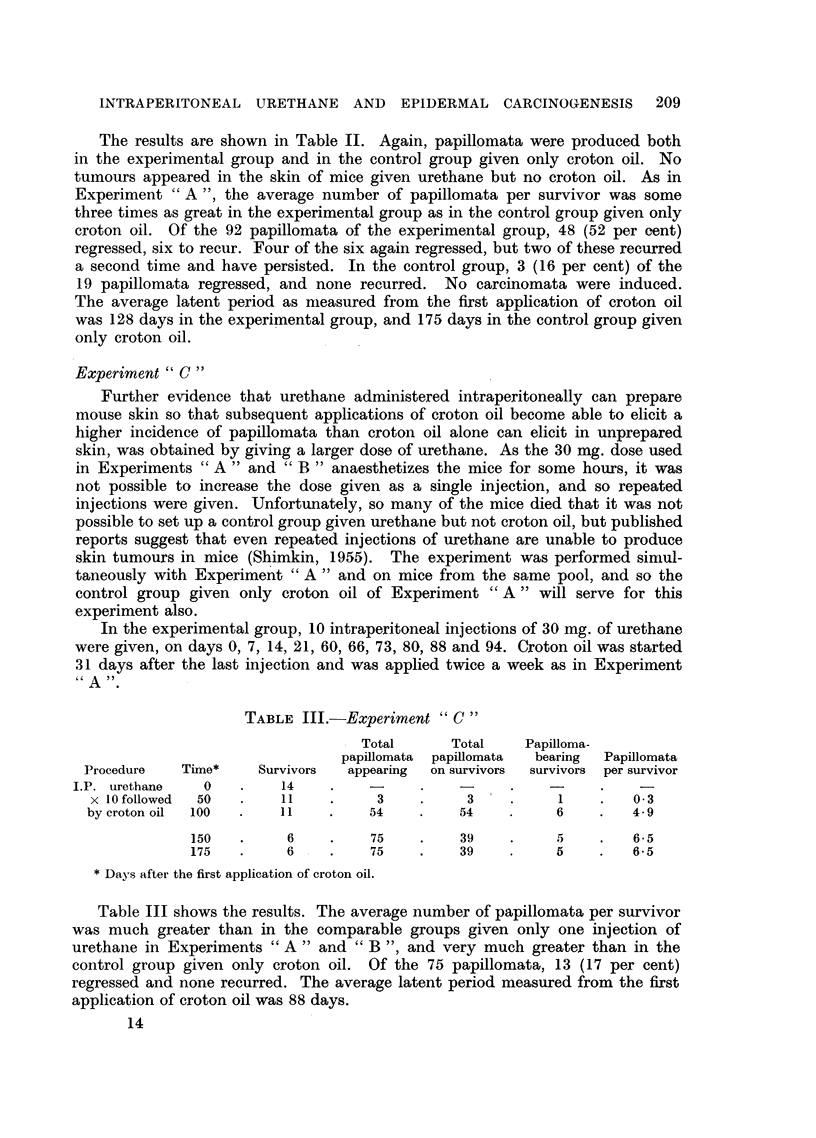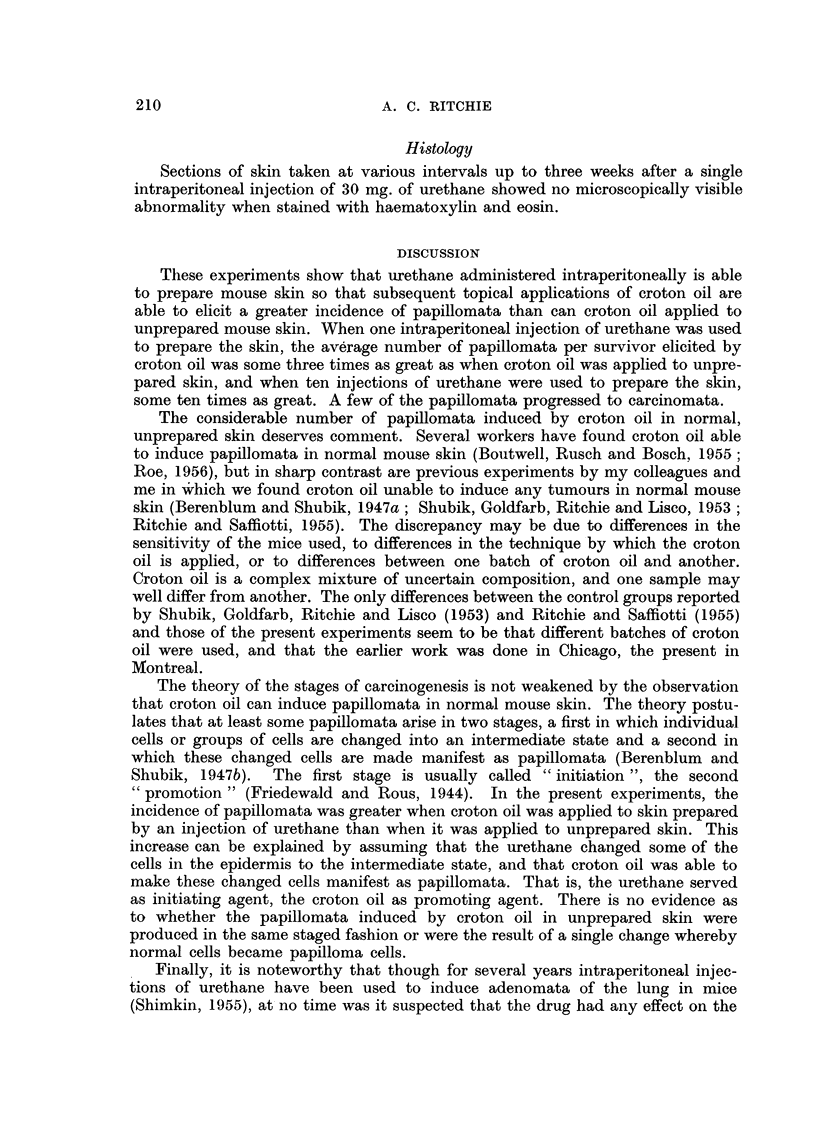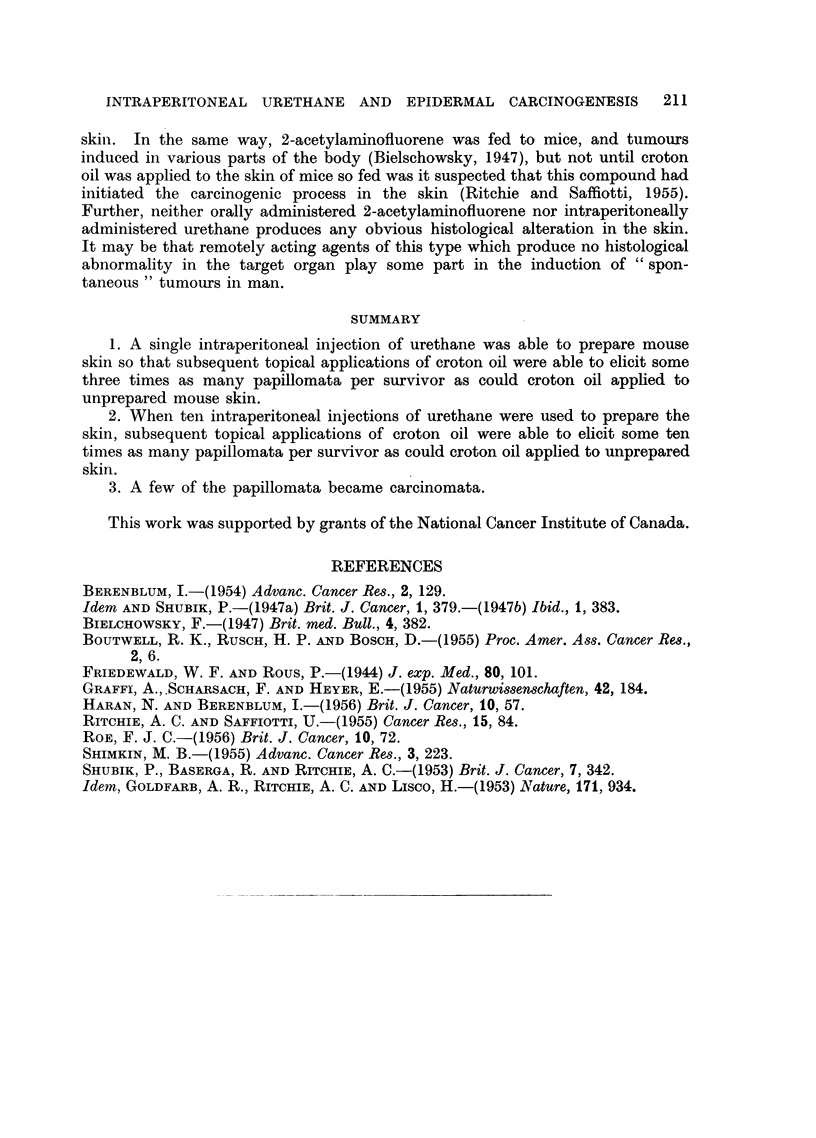# Epidermal Carcinogenesis in the Mouse by Intraperitoneally Administered Urethane Followed by Repeated Applications of Croton Oil

**DOI:** 10.1038/bjc.1957.28

**Published:** 1957-06

**Authors:** A. C. Ritchie


### EPIDERMAL CARCINOGENESIS IN THE MOUSE BY INTRA-PERITONEALLY ADMINISTERED URETHANE FOLLOWED BY REPEATED APPLICATIONS OF CROTON OIL

A. C. RITCHIE

*From the Department of Pathology, Pathological Institute, McGill University, Montreal, Canada*

Received for publication February 25, 1957

EPIDERMAL carcinogenesis in the mouse has been divided into two stages (Berenblum, 1954). In the first, the skin is prepared so that in the second a suitable non-carcinogenic or weakly carcinogenic agent is able to elicit a high incidence of epidermal tumours in the prepared skin. Most of the agents found able to prepare the skin in this way have been applied topically, but in three instances the agent was given by another route. 2-Acetylaminofluorene given by mouth (Ritchie and Saffiotti, 1955), 9,10-dimethyl-1,2-benzanthracene given orally, intraperitoneally or intravenously (Graffi, Scharsach and Heyer, 1955) and urethane given by mouth (Haran and Berenblum, 1956) have all been reported able to prepare mouse skin in this way. The present paper describes a fourth manner in which mouse skin can be prepared in this way by an agent which is not applied topically. Intraperitoneally administered urethane has been found adequate to prepare mouse skin so that subsequent topical applications of croton oil are able to elicit a higher incidence of papillomata than croton oil can elicit in unprepared skin.

### METHOD

Virgin, adult, female mice of the Swiss strain obtained from the Roscoe B. Jackson Memorial Laboratory, Bar Harbor, Maine, were used. In each experiment, the mice of the experimental and control groups were drawn at random from a common pool, all animals weighing about 20 g. The mice were fed Rockland Mouse Diet, being given food and water *ad libitum*. They were weighed weekly, but no abnormal alteration in their weight was noted at any time during the experiments. " Urethan Merck " (Ethyl carbamate) U.S.P. (lot 6839) was injected as a 10 per cent w/v solution in distilled water, 0·3 ml. being given intraperitoneally. 01. croton. B.P.C. (Boot's Pure Drug Co., batch 5596A) was applied as a 5 per cent v/v solution in heavy liquid paraffin B.P. (British Drug Houses (Canada) lot 12578), being spread sparely over the whole back with a fine camel's hair brush. The painted area was kept shorn with electric clippers or scissors. Control mice in groups not painted with croton oil were clipped in the same way as those painted. The mice were examined twice a week, and any changes in the number, size, type or position of the tumours charted on special diagrams.

### EXPERIMENTAL

The skin tumours all arose within the area painted with croton oil. All were papillomata when they first appeared, and most remained sessile papillomata of the type described by Shubik, Baserga and Ritchie (1953). A few progressed to become pedunculated papillomata and a few became carcinomata. No carcinoma arose except from a pre-existing papilloma. As is usual in experiments in which croton oil is applied to suitably prepared mouse skin, many of the papillomata regressed, perhaps to recur and persist or to recur and again regress. In calculating the results, no papilloma has been included unless it persisted for at least 10 days. The tables record the total number of papillomata which appeared. No deduction has been made for papillomata which regressed, or for papillomata present on mice which died. Papillomata which recurred have not been counted as new tumours. Details as to the number of regressions and recurrences will be given in the text. In addition, the tables record the total number of papillomata on the survivors and, to facilitate comparison between groups, the average number of papillomata per survivor, a figure obtained by dividing the total number of papillomata which had appeared on the survivors by the number of survivors. Once again, no deduction has been made for papillomata which regressed and papillomata recurring have not been counted as new tumours. The latent period of a papilloma has been taken to be the period between the first application of croton oil and its first appearance. In calculating the average latent period, every papilloma has been considered, not only those which were the first to appear on their host. For papillomata which regressed and subsequently recurred, the latent period has been taken to be the time up to their first appearance.

*Experiment " A "*

In the experimental group, 20 mice were given a single intraperitoneal injection of 30 mg. of urethane. Applications of croton oil were begun 31 days later and were continued twice a week until the end of the experiment. A control group of 49 mice was given no urethane, but was painted with croton oil just as was the experimental group.

Table I shows the results. Many papillomata were produced in both the experimental and control groups, but it is apparent that the average number of papillomata per survivor was nearly three times as great in the experimental group as in the control. Of the 50 papillomata in the experimental group, 21 (42 per cent) regressed. Six of the 21 recurred, but one again regressed. In the control group, 17 of the 63 (27 per cent) papillomata regressed. Three of the 17 recurred, one to regress a second time, recur again, and regress a third time. In the experimental group, three of the papillomata became carcinomata, the first carcinoma appearing on day 297. In the control group, one carcinoma appeared on day 364. In the experimental group, the average latent period as measured from the first application of croton oil was 121 days, in the control group 210 days.

*Experiment " B "*

To confirm the findings of Experiment " A ", the work was repeated. An experimental group of 30 mice was given a single intraperitoneal injection of 30 mg. of urethane, and 31 days later applications of croton oil were begun and were continued twice a week. A control group of 19 mice was painted with croton oil but received no urethane, and an additional control group of 20 mice was given urethane but no croton oil.

TABLE I.—*Experiment "A"*

| Procedure | Time* | Survivors | Total papillomata appearing | Total papillomata on survivors | Papilloma-bearing survivors | Papillomata per survivor |
|---|---|---|---|---|---|---|
| I.P. urethane followed by croton oil | 0 | 20 | — | — | — | — |
| | 50 | 20 | — | — | — | — |
| | 100 | 16 | 25 | 25 | 9 | 1·6 |
| | 150 | 16 | 37 | 37 | 10 | 2·3 |
| | 200 | 16 | 44 | 44 | 11 | 2·8 |
| | 250 | 14 | 48 | 37 | 10 | 2·6 |
| | 300 | 11 | 48 | 37 | 10 | 3·4 |
| | 350 | 9 | 50 | 32 | 8 | 3·6 |
| | 375 | 6 | 50 | 26 | 6 | 4·3 |
| Croton oil only | 0 | 49 | — | — | — | — |
| | 50 | 46 | — | — | — | — |
| | 100 | 46 | 6 | 6 | 3 | 0·1 |
| | 150 | 45 | 18 | 18 | 7 | 0·4 |
| | 200 | 43 | 40 | 29 | 13 | 0·7 |
| | 250 | 41 | 47 | 36 | 16 | 0·9 |
| | 300 | 39 | 53 | 42 | 19 | 1·1 |
| | 350 | 36 | 58 | 47 | 18 | 1·3 |
| | 375 | 34 | 63 | 52 | 17 | 1·5 |

* Days after the first application of croton oil.

TABLE II.—*Experiment "B"*

| Procedure | Time* | Survivors | Total papillomata appearing | Total papillomata on survivors | Papilloma-bearing survivors | Papillomata per survivor |
|---|---|---|---|---|---|---|
| I.P. urethane followed by croton oil | 0 | 30 | — | — | — | — |
| | 50 | 30 | — | — | — | — |
| | 100 | 30 | 46 | 46 | 19 | 1·5 |
| | 150 | 29 | 60 | 60 | 20 | 2·1 |
| | 200 | 28 | 75 | 73 | 21 | 2·6 |
| | 250 | 25 | 92 | 90 | 20 | 3·6 |
| Croton oil only | 0 | 19 | — | — | — | — |
| | 50 | 19 | — | — | — | — |
| | 100 | 18 | 3 | 3 | 2 | 0·2 |
| | 150 | 18 | 7 | 7 | 4 | 0·4 |
| | 200 | 16 | 11 | 11 | 6 | 0·7 |
| | 250 | 14 | 19 | 19 | 7 | 1·4 |
| Urethane only | 0 | 20 | — | — | — | — |
| | 50 | 20 | — | — | — | — |
| | 100 | 20 | — | — | — | — |
| | 150 | 19 | — | — | — | — |
| | 200 | 16 | — | — | — | — |
| | 250 | 14 | — | — | — | — |

* Days after first application of croton oil or in group given urethane only from 31 days after the injection.

The results are shown in Table II. Again, papillomata were produced both in the experimental group and in the control group given only croton oil. No tumours appeared in the skin of mice given urethane but no croton oil. As in Experiment " A ", the average number of papillomata per survivor was some three times as great in the experimental group as in the control group given only croton oil. Of the 92 papillomata of the experimental group, 48 (52 per cent) regressed, six to recur. Four of the six again regressed, but two of these recurred a second time and have persisted. In the control group, 3 (16 per cent) of the 19 papillomata regressed, and none recurred. No carcinomata were induced. The average latent period as measured from the first application of croton oil was 128 days in the experimental group, and 175 days in the control group given only croton oil.

*Experiment " C "*

Further evidence that urethane administered intraperitoneally can prepare mouse skin so that subsequent applications of croton oil become able to elicit a higher incidence of papillomata than croton oil alone can elicit in unprepared skin, was obtained by giving a larger dose of urethane. As the 30 mg. dose used in Experiments " A " and " B " anaesthetizes the mice for some hours, it was not possible to increase the dose given as a single injection, and so repeated injections were given. Unfortunately, so many of the mice died that it was not possible to set up a control group given urethane but not croton oil, but published reports suggest that even repeated injections of urethane are unable to produce skin tumours in mice (Shimkin, 1955). The experiment was performed simultaneously with Experiment " A " and on mice from the same pool, and so the control group given only croton oil of Experiment " A " will serve for this experiment also.

In the experimental group, 10 intraperitoneal injections of 30 mg. of urethane were given, on days 0, 7, 14, 21, 60, 66, 73, 80, 88 and 94. Croton oil was started 31 days after the last injection and was applied twice a week as in Experiment " A ".

TABLE III.—*Experiment " C "*

| Procedure | Time* | Survivors | Total papillomata appearing | Total papillomata on survivors | Papilloma-bearing survivors | Papillomata per survivor |
|---|---|---|---|---|---|---|
| I.P. urethane | 0 | 14 | — | — | — | — |
| × 10 followed | 50 | 11 | 3 | 3 | 1 | 0·3 |
| by croton oil | 100 | 11 | 54 | 54 | 6 | 4·9 |
| | 150 | 6 | 75 | 39 | 5 | 6·5 |
| | 175 | 6 | 75 | 39 | 5 | 6·5 |

* Days after the first application of croton oil.

Table III shows the results. The average number of papillomata per survivor was much greater than in the comparable groups given only one injection of urethane in Experiments " A " and " B ", and very much greater than in the control group given only croton oil. Of the 75 papillomata, 13 (17 per cent) regressed and none recurred. The average latent period measured from the first application of croton oil was 88 days.

*Histology*

Sections of skin taken at various intervals up to three weeks after a single intraperitoneal injection of 30 mg. of urethane showed no microscopically visible abnormality when stained with haematoxylin and eosin.

### DISCUSSION

These experiments show that urethane administered intraperitoneally is able to prepare mouse skin so that subsequent topical applications of croton oil are able to elicit a greater incidence of papillomata than can croton oil applied to unprepared mouse skin. When one intraperitoneal injection of urethane was used to prepare the skin, the average number of papillomata per survivor elicited by croton oil was some three times as great as when croton oil was applied to unprepared skin, and when ten injections of urethane were used to prepare the skin, some ten times as great. A few of the papillomata progressed to carcinomata.

The considerable number of papillomata induced by croton oil in normal, unprepared skin deserves comment. Several workers have found croton oil able to induce papillomata in normal mouse skin (Boutwell, Rusch and Bosch, 1955 ; Roe, 1956), but in sharp contrast are previous experiments by my colleagues and me in which we found croton oil unable to induce any tumours in normal mouse skin (Berenblum and Shubik, 1947*a* ; Shubik, Goldfarb, Ritchie and Lisco, 1953 ; Ritchie and Saffiotti, 1955). The discrepancy may be due to differences in the sensitivity of the mice used, to differences in the technique by which the croton oil is applied, or to differences between one batch of croton oil and another. Croton oil is a complex mixture of uncertain composition, and one sample may well differ from another. The only differences between the control groups reported by Shubik, Goldfarb, Ritchie and Lisco (1953) and Ritchie and Saffiotti (1955) and those of the present experiments seem to be that different batches of croton oil were used, and that the earlier work was done in Chicago, the present in Montreal.

The theory of the stages of carcinogenesis is not weakened by the observation that croton oil can induce papillomata in normal mouse skin. The theory postulates that at least some papillomata arise in two stages, a first in which individual cells or groups of cells are changed into an intermediate state and a second in which these changed cells are made manifest as papillomata (Berenblum and Shubik, 1947*b*). The first stage is usually called " initiation ", the second " promotion " (Friedewald and Rous, 1944). In the present experiments, the incidence of papillomata was greater when croton oil was applied to skin prepared by an injection of urethane than when it was applied to unprepared skin. This increase can be explained by assuming that the urethane changed some of the cells in the epidermis to the intermediate state, and that croton oil was able to make these changed cells manifest as papillomata. That is, the urethane served as initiating agent, the croton oil as promoting agent. There is no evidence as to whether the papillomata induced by croton oil in unprepared skin were produced in the same staged fashion or were the result of a single change whereby normal cells became papilloma cells.

Finally, it is noteworthy that though for several years intraperitoneal injections of urethane have been used to induce adenomata of the lung in mice (Shimkin, 1955), at no time was it suspected that the drug had any effect on the skin. In the same way, 2-acetylaminofluorene was fed to mice, and tumours induced in various parts of the body (Bielschowsky, 1947), but not until croton oil was applied to the skin of mice so fed was it suspected that this compound had initiated the carcinogenic process in the skin (Ritchie and Saffiotti, 1955). Further, neither orally administered 2-acetylaminofluorene nor intraperitoneally administered urethane produces any obvious histological alteration in the skin. It may be that remotely acting agents of this type which produce no histological abnormality in the target organ play some part in the induction of "spontaneous" tumours in man.

### SUMMARY

1. A single intraperitoneal injection of urethane was able to prepare mouse skin so that subsequent topical applications of croton oil were able to elicit some three times as many papillomata per survivor as could croton oil applied to unprepared mouse skin.

2. When ten intraperitoneal injections of urethane were used to prepare the skin, subsequent topical applications of croton oil were able to elicit some ten times as many papillomata per survivor as could croton oil applied to unprepared skin.

3. A few of the papillomata became carcinomata.

This work was supported by grants of the National Cancer Institute of Canada.

### REFERENCES

BERENBLUM, I.—(1954) *Advanc. Cancer Res.*, **2**, 129.

*Idem* AND SHUBIK, P.—(1947a) *Brit. J. Cancer*, **1**, 379.—(1947*b*) *Ibid.*, **1**, 383.

BIELCHOWSKY, F.—(1947) *Brit. med. Bull.*, **4**, 382.

BOUTWELL, R. K., RUSCH, H. P. AND BOSCH, D.—(1955) *Proc. Amer. Ass. Cancer Res.*, **2**, 6.

FRIEDEWALD, W. F. AND ROUS, P.—(1944) *J. exp. Med.*, **80**, 101.

GRAFFI, A., SCHARSACH, F. AND HEYER, E.—(1955) *Naturwissenschaften*, **42**, 184.

HARAN, N. AND BERENBLUM, I.—(1956) *Brit. J. Cancer*, **10**, 57.

RITCHIE, A. C. AND SAFFIOTTI, U.—(1955) *Cancer Res.*, **15**, 84.

ROE, F. J. C.—(1956) *Brit. J. Cancer*, **10**, 72.

SHIMKIN, M. B.—(1955) *Advanc. Cancer Res.*, **3**, 223.

SHUBIK, P., BASERGA, R. AND RITCHIE, A. C.—(1953) *Brit. J. Cancer*, **7**, 342.

*Idem*, GOLDFARB, A. R., RITCHIE, A. C. AND LISCO, H.—(1953) *Nature*, **171**, 934.